# RNA-Seq profiling of circular RNA in human lung adenocarcinoma and squamous cell carcinoma

**DOI:** 10.1186/s12943-019-1061-8

**Published:** 2019-09-04

**Authors:** Chengdi Wang, Shuangyan Tan, Wen-Rong Liu, Qian Lei, Wenliang Qiao, Yangping Wu, Xiaoqi Liu, Wei Cheng, Yu-Quan Wei, Yong Peng, Weimin Li

**Affiliations:** 1 Department of Respiratory and Critical Care Medicine, West China Medical School/West China Hospital, Sichuan University, Chengdu, 610041 China; 2 State Key Laboratory of Biotherapy and Cancer Center, West China Hospital, Sichuan University, Chengdu, 610041 China; 3 Department of Targeted Tracer Research and Development Laboratory, West China Hospital, Sichuan University, Chengdu, 610041 China; 4 Lung Cancer Center, West China Hospital, Sichuan University, Chengdu, 610041 China

**Keywords:** Circular RNAs, Lung adenocarcinoma, Lung squamous carcinoma, circRNA sequencing

## Abstract

Emerging evidences demonstrate that circular RNAs (circRNAs) are abnormally expressed in tumors and could serve as prognostic markers for cancers. However, the expression patterns and clinical implications of circRNAs in non-small cell lung cancer (NSCLC) remain obscure. In this study, we profiled circRNA expressions in 10 pairs of lung adenocarcinoma (LUAD) and squamous cell carcinoma (LUSC) after ribosomal RNA-depletion and RNase R digestion to enrich circRNAs. Combining five circRNA computational programs, we found that LUAD and LUSC not only share common expression patterns, but also exhibit distinct circRNA expression signatures. Moreover, the Receiver Operating Characteristic (ROC) curve analysis indicated that hsa_circ_0077837 and hsa_circ_0001821 could serve as potential biomarkers for both LUAD and LUSC, while hsa_circ_0001073 and hsa_circ_0001495 could be diagnostic/subtyping marker for LUAD and LUSC, respectively. Therefore, our findings highlight the important diagnostic potential of circRNAs in NSCLC.

**Electronic supplementary material:**

The online version of this article (10.1186/s12943-019-1061-8) contains supplementary material, which is available to authorized users.

## Main text

Lung cancer is the leading cause of malignancy-related mortality. Approximately 85% of lung cancer belong to non-small cell lung cancer (NSCLC), including lung adenocarcinoma (LUAD) and lung squamous cell carcinoma (LUSC) [1]. Despite recent advances in diagnostic and therapeutic approaches, the 5-year overall survival for NSCLC still remains poor [2]. To improve the NSCLC diagnosis and prognosis, it is in an urgent need to elucidate molecular mechanisms underlying NSCLC and identify their new reliable biomarkers and therapeutic targets.

Circular RNAs (circRNAs) are a special class of endogenous RNAs with covalently closed loop structure, conferring them remarkable tolerance to exonucleases. Accumulating evidences demonstrate the involvement of circRNAs in normal physiological processes and development of various diseases including lung cancer [3]. Moreover, circRNAs were reported to have diagnostic and prognostic potential for NSCLC. For instance, F-circEA generated from EML4-ALK fusion gene could serve as a promising liquid biopsy biomarker for the diagnosis of NSCLC patients harboring this fusion gene [4]. However, the expression profile and possible roles of circRNAs in NSCLC largely remain unclear.

In this study, we employed circRNA sequencing and five circRNA computational programs to identify differentially expressed circRNAs in LUAD and LUSC tissues. Our results showed that LUAD and LUSC not only share common expression patterns of certain circRNAs, but also exhibited distinct circRNA expressions. Moreover, certain circRNAs could serve as diagnostic biomarkers for NSCLC.

## Results and discussion

### CircRNA profiling in human lung tumors and their adjacent normal tissues

**Fig. 1:**
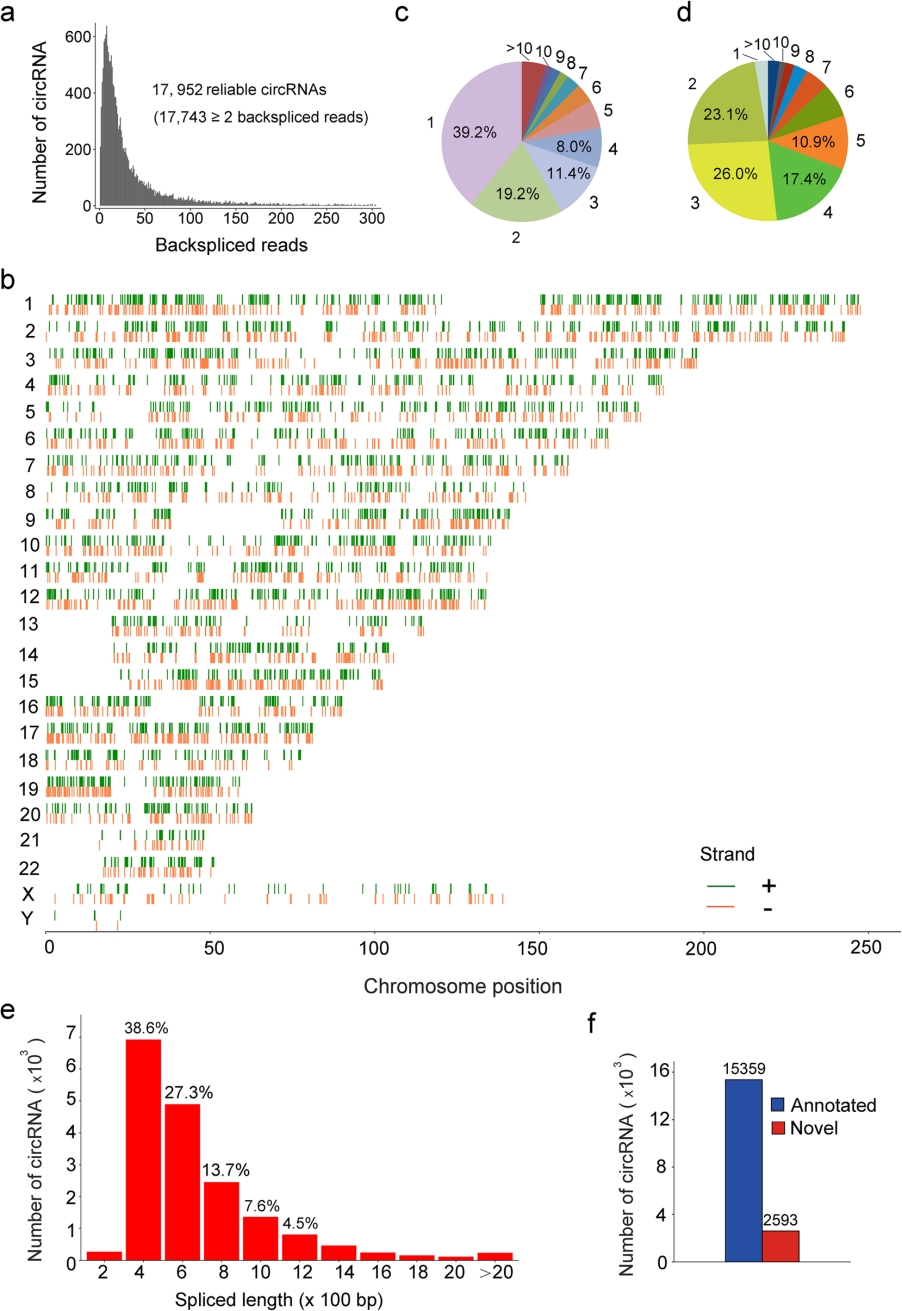
Characterization of circRNAs identified in lung tumors and their adjacent normal tissues. **a** The number of circRNAs and back-spliced reads identified in lung tumors and normal tissues. **b** The chromosome distribution of total identified circRNAs in lung tissues. **c** Number of circRNAs produced from one gene. **d** Exon numbers of identified circRNAs. **e** The length distribution of identified circRNAs. **f** Comparison of circRNAs identified in this study and circBase

Given that circRNA is of low abundance and high resistance to RNase R, we treated ribosomal RNA-depleted total RNAs with RNase R to degrade linear RNAs and enrich circRNAs (Additional file 1). This procedure could greatly reduce the background noise and promote the reliability and accuracy of circRNA identification. Following RNase R-digestion, 10 pairs of RNA samples from NSCLC tumors and their corresponding normal tissues were subjected to high-throughput RNA sequencing (Additional file 2: Table S1–2). The sequencing dataset was analyzed utilizing 5 programs (CIRCexplorer2, circRNA_finder, CIRI2, find_circ and MapSplice) to comprehensively screen reliable circRNAs. A total of 17,952 circRNAs were found across all 5 programs, and nearly 98.84% of them contained at least 2 unique back-spliced reads (Fig. 1a, Additional file 3: Figure S1a). It is observed that these overlapped circRNAs ubiquitously located in whole genomic regions (Fig. 1b).

Since one gene could generate multiple circRNAs through an alternative back-splicing mechanism [3], we investigated to what extent the alternative back-splicing contributes to circRNA diversity in lung tissues. As shown in Fig. 1c, nearly 60.8% host genes corresponding to the screened-out circRNAs can produce at least 2 circRNAs. For example, the YAP1 gene yields three distinct circRNAs through two mRNA transcripts (Additional file 3: Figure S1b). Strikingly, some genes could generate more than 10 circRNAs (Additional file 2: Table S3). Although ubiquitously locating across whole genomic regions, most circRNAs were back-spliced from exonic region, mainly (77.4%) consisting of 2–5 exons (Fig. 1d; Additional file 2: Table S4). Additionally, the length of most circRNAs (91.7%) is between 200 and 1200 nucleotides (Fig. 1e; Additional file 2: Table S5). Among these screened-out circRNAs, 15,359 are already recorded in circBase database that contains 140,790 human circRNAs [5], and thus 2593 are considered novel (Fig. 1f).

### Identification and validation of differentially expressed circRNAs in LUAD and LUSC tissues

**Fig. 2:**
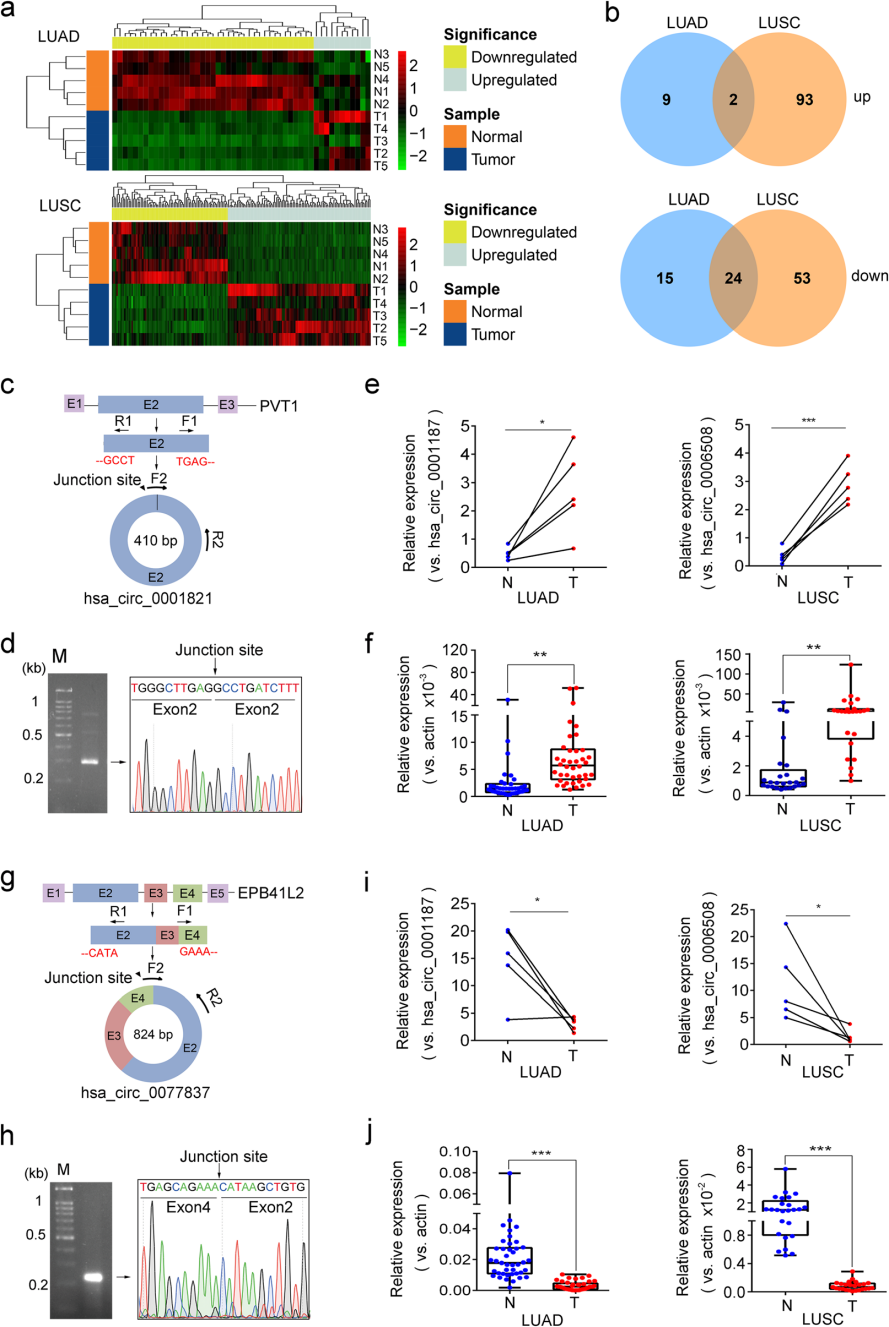
Identification and validation of differentially expressed circRNAs in LUAD and LUSC. **a** Hierarchical clustering heatmap of dysregulated circRNAs between LUAD or LUSC and their adjacent normal tissues. **b** Comparison of differentially expressed circRNAs identified in LUAD and LUSC tissues. **c**, **g** Schematic diagram of hsa_circ_0001821(**c**) and hsa_circ_0077837(**g**). **d**, **h** Agarose gel electrophoresis and Sanger sequencing of RT-PCR products of hsa_circ_0001821(**d**) and hsa_circ_0077837(**h**). **e-f**, **i-j** Relative expression of hsa_circ_0001821 (**e**, **f**) and hsa_circ_0077837 (**i**, **j**) in LUAD and LUSC tissues for circRNA sequencing and in another independent cohort of NSCLC patients’ samples

Comparing circRNA expression in NSCLC tumors with those in their respective adjacent normal tissues, 50 circRNAs were found to be differentially expressed in LUAD tissues with corrected *p* value ≤0.05 and fold change ≥2 (Fig. 2a, Additional file 3: Figure S2a). Among them, the upregulation of hsa_circ_0002360 in LUAD was validated by Sanger sequencing and qPCR (Additional file 3: Figure S2c-f), consistent with the previous report [6]. Using the same stringent criteria, 172 circRNAs were observed to have significantly differential expressions in LUSC tissues (Fig. 2a, Additional file 3: Figure S2b) and 26 circRNAs differentially expressed in both LUAD and LUSC tissues (Fig. 2b). Such aberrant circRNA expression could be caused by chromosomal amplification/deletion, transcriptional change or abnormal circRNA biogenesis [7–9]. Together, LUAD and LUSC tissues not only share common differential expressions of some circRNAs, but also exhibit distinct circRNA expressions, implying that the former circRNAs have common functions in LUAD and LUSC and the latter circRNAs play NSCLC subtype-specific roles.

Four differentially expressed circRNAs analyzed as above were selected for further validation because of their high abundance and large fold changes in expression. Among them, hsa_circ_0001073 and hsa_circ_0001495 had altered expressions only in LUAD and LUSC, respectively, while hsa_circ_0077837 and hsa_circ_0001821 had significantly differential expression in both subtypes. For validation, PCRs were conducted for each circRNA using the specific divergent primer sets of F1/R1 spanning the respective junction sites (Fig. 2c and g, Additional file 3: Figure S3a and g). The Sanger sequencing results of PCR products confirmed the existence of back-spliced junction sites of all 4 selected circRNAs (Fig. 2d and h, Additional file 3: Figure S3b and h), which are consistent with the circBase. Then we performed qPCR for each circRNA using different divergent primer sets of F2/R2 with F2 crossing respective circRNA junction site. The upregulation of hsa_circ_0001821 and the downregulation of hsa_circ_0077837 in both LUAD and LUSC tissues were firstly validated in the profiling samples (Fig. 2e and i) and then confirmed in an independent cohort of patient samples (Fig. 2f and j). Moreover, qPCR data using another divergent primer sets of F3/R3 with one primer crossing the junction site (Additional file 2: Table S6) further verified the differential expressions of hsa_circ_0001821 and hsa_circ_0077837 in both LUAD and LUSC tissues (Additional file 3: Figure S3 m and n), implying they could serve as oncogene or tumor suppressor during NSCLC tumorigenesis. Increasing studies revealed the important functions of circRNAs in tumor development. For example, hsa_circ_0001821 (circPVT1) plays an oncogenic role in gastric cancer [10]. Our observations that hsa_circ_0001821 is highly expressed in both LUAD and LUSC tissues suggest its potential role in NSCLC progression as well.

Similarly, hsa_circ_0001073 and hsa_circ_0001495 were confirmed to abnormally express in NSCLC tumors in subtype-specific patterns, i.e., hsa_circ_0001073 was downregulated only in LUAD tissues while hsa_circ_0001495 exhibited higher expression only in LUSC tissues (Additional file 3: Figure S3c-f, i-l). The distinct, NSCLC subtype-specific dysregulations of these circRNAs suggest their subtype-specific biological functions.

### Potential biomarker of circRNA for NSCLC diagnosis

**Fig. 3:**
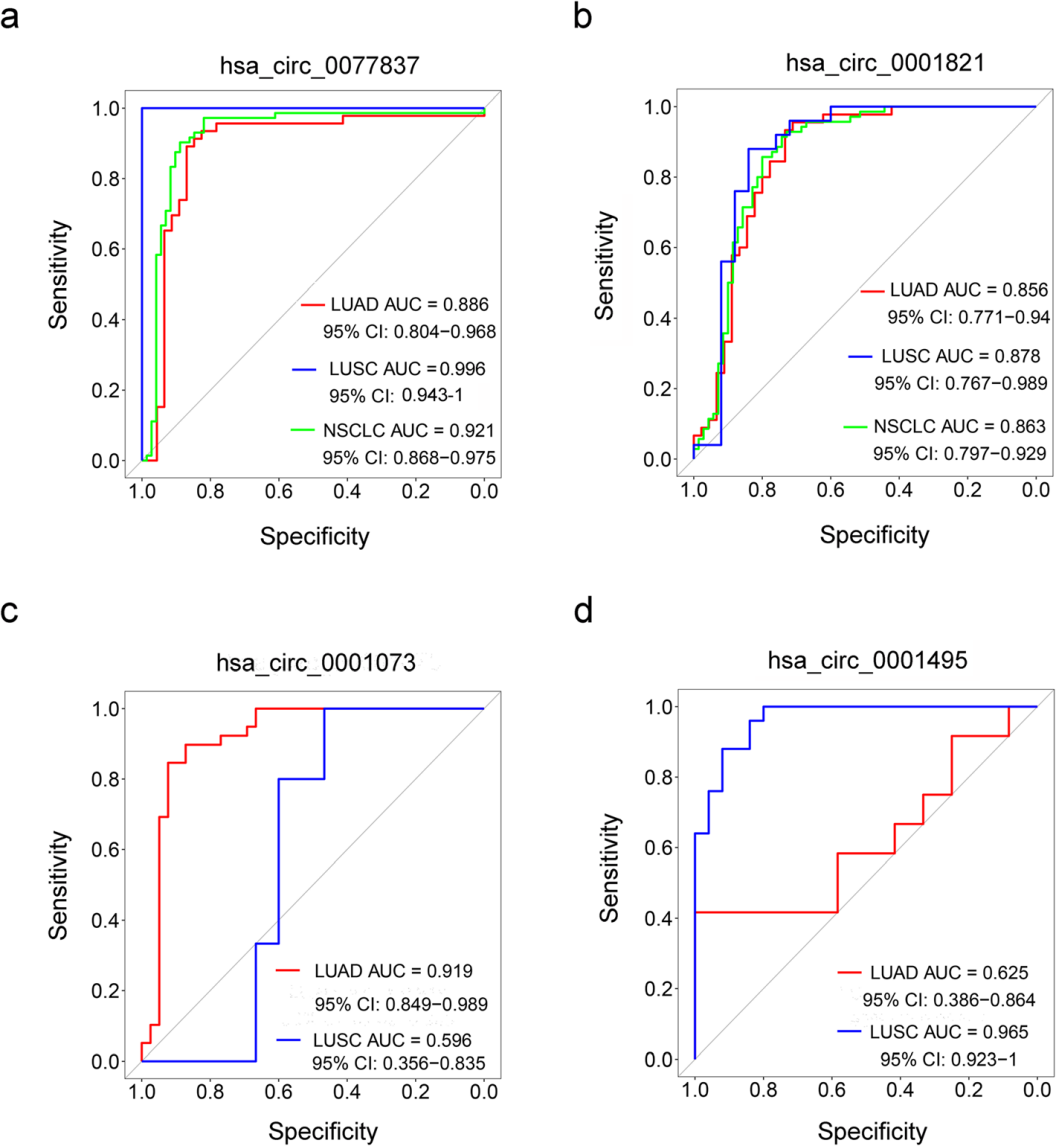
ROC analyses of differentially expressed circRNAs in LUAD and LUSC. **a-d** ROC curve analyses of hsa_circ_0077837 (**a**), hsa_circ_0001821 (**b**), hsa_circ_0001073 (**c**) and hsa_circ_0001495 (**d**) for NSCLC, LUAD or LUSC diagnosis

To explore the diagnostic potential of the above selected circRNAs, we performed the Receiver Operating Characteristic (ROC) curve analysis. As shown in Fig. 3a and b, the area under the curve (AUC) to discriminate NSCLC from normal tissues was 0.921 (95% CI: 0.868–0.975) for hsa_circ_0077837 and 0.863 (95% CI: 0.797–0.929) for hsa_circ_0001821, suggesting the high diagnostic potential of these two circRNAs in NSCLC patients. However, these two circRNAs are unable to distinguish LUAD and LUSC subtypes. On the other hand, the AUC was 0.919 for hsa_circ_0001073 in LUAD tissues (Fig. 3c) and 0.965 for hsa_circ_0001495 in LUSC tissues (Fig. 3d), implying that hsa_circ_0001073 and hsa_circ_0001495, which have been shown as above to abnormally express in NSCLC subtype-specific patterns, could serve as diagnostic biomarkers to predict LUAD and LUSC, respectively.

## Conclusions

In this study, we profiled circRNA expressions in NSCLC including LUAD and LUSC tumors and their adjacent normal tissues, and found that LUAD and LUSC tissues not only share the common differential expression patterns, but also hold distinct circRNA expression signatures. Moreover, ROC analyses demonstrate that hsa_circ_0077837 and hsa_circ_0001821 have the diagnostic potential for NSCLC patients, while hsa_circ_0001073 and hsa_circ_0001495 could act as biomarkers for NSCLC pathological subtyping.

## Additional files

Additional file 1: Supplementary materials and methods. (DOCX 46 kb)

Additional file 2: **Table S1.** Clinical characteristics of LUAD patients enrolled in this study. **Table S2.** Clinical characteristics of LUSC patients enrolled in this study. **Table S3.** The number of circRNAs produced from one gene. **Table S4.** Exon numbers of identified circRNAs. **Table S5.** The length distribution of identified circRNAs. **Table S6.** Information of primers used in this study. (DOCX 31 kb)

Additional file 3: **Figure S1.** Identification of circRNAs expressed in lung tumors and their adjacent normal tissues. **Figure S2.** Identification of differentially expressed circRNAs in LUAD and LUSC tissues. **Figure S3.** Validation of four selected circRNAs in LUAD and LUSC tissues. (DOCX 1305 kb)

## Data Availability

All the data obtained and/or analyzed during the current study were available from the corresponding authors on reasonable request.

## Abbreviations

AUC: Area under the curve
CI: Confidence interval
circRNAs: circular RNAs
LUAD: Lung adenocarcinoma
LUSC: Lung squamous carcinoma
NSCLC: Non-small cell lung cancer
qPCR: quantitative real-time polymerase chain reaction
ROC: Receiver operating characteristic
RT-PCR: Reverse transcription PCR
