# Supplementary material for: RNA-Seq profiling of circular RNA in human lung adenocarcinoma and squamous cell carcinoma
Source: Mol Cancer. 2019 Sep 4;18:134. doi: 10.1186/s12943-019-1061-8 (PMC6724331; doi:10.1186/s12943-019-1061-8)
Supplement: Supplementary file 1 — Supplementary materials and methods. (DOCX 46 kb) [file 12943_2019_1061_MOESM1_ESM.docx]

# Supplementary Materials and Methods

**Human lung tumors and their adjacent normal tissues**

For circRNA sequencing, totally 10 pairs of fresh NSCLC tumors and their adjacent normal tissues from 5 lung adenocarcinoma (LUAD) patients and 5 lung squamous cell carcinoma (LUSC) patients were collected at West China Hospital, Sichuan University. For qPCR measurement of circRNA expressions, a total of 67 pairs of NSCLC tumors and adjacent normal tissues from the 42 LUAD patients and 25 LUSC patients were obtained from West China Biobanks, Department of Clinical Research Management, West China Hospital, Sichuan University. The detailed clinical characteristics of patients enrolled in this study were summarized in Table S1 and Table S2 (Additional file 2). This study was approved by the Ethics Committee of West China Hospital and written informed consent was obtained from the patients.

**CircRNA sequencing**

Total RNAs were extracted from patients’ tissues with TRIzol^TM^ reagent (Invitrogen, USA) according to the manufacturer’s instructions. RNA integrity and DNA contamination were assessed using electrophoresis on a denaturing agarose gel. The RNA samples with RNA integrity number (RIN) ≥7.0 and a 28S:18S ratio ≥1.5 were chosen for subsequent treatment. After removal of ribosomal RNAs by Epicentre Ribo-zero^TM^ rRNA Removal Kit (Epicentre Technologies, USA) and digestion of linear RNAs with RNase R (Epicentre Technologies, USA), the resultant RNAs were subjected to generating sequencing library by NEBNext^®^ Ultra^TM^ Directional RNA Library Prep Kit for Illumina^®^ (NEB, USA). Then PCR was performed with Phusion High-Fidelity DNA polymerase, Universal PCR primers and Index (X) Primer. At last, products were purified (AMPure XP system) and library quality was assessed on the Agilent Bioanalyzer 2100 system

**CircRNA identification**

Five programs including CIRCexplorer2 (v2.3.3) [[1](#_ENREF_1)], circRNA_finder (v1.1) [[2](#_ENREF_2)], CIRI2 (v2.0.6) [[3](#_ENREF_3)], find_circ (v1.2) [[4](#_ENREF_4)] and MapSplice (v2.2.1) [[5](#_ENREF_5)] were utilized to identify circRNA with their suggested settings. Reference genome (GRCh37.p13 version) and gene annotation (gencode.v19 version) required for circRNA identification were downloaded from GENCODE database. After assessment of the quality of RNA-seq data by FastQC, the clean reads were obtained by removing low quality reads and mapped to the reference genome using the designated reads aligner recommended by the corresponding program. Prediction of circRNA was then conducted by each program with default parameters, and the circRNAs predicted by all of five programs were considered as high confidential circRNA candidates for further analysis. Lastly, the overlapped circRNAs were annotated with circBase (140,790 human circRNAs) [[6](#_ENREF_6)] database to determine whether these circRNAs are novel or not.

**Differential expression of circRNAs in LUAD and LUSC**

To quantify overlapped circRNAs, the number of back-spliced junction reads of each circRNA was extracted and combined. Then circRNA abundance was determined and scaled to RPM (Reads Per Million mapped reads) as previously described [[7](#_ENREF_7)]. The circRNA with RPM greater than 0.1 in at least 10% samples were kept for differential expression analysis. The R package DESeq2 [[8](#_ENREF_8)] was used to identify differentially expressed circRNAs between normal and tumor groups. CircRNAs with the absolute value of fold-change greater than 2 and adjusted *p* value less than 0.05 were determined as significantly differentially expression circRNAs. Next, differential expression analysis results from five programs were merged and managed. Finally, circRNAs with consistent differential expression pattern in five programs were kept.

**Validation and quantification of circRNA**

Total RNAs were reverse transcribed by random primers using the M-MLV Reverse Transcriptase Kit (Life Technologies, USA), and the resultant cDNAs were subjected to PCR reactions using Phanta**^®^** Max Super-Fidelity DNA Polymerase (Vazyme, China) and specific divergent primers, which were listed in Table S6 (Additional file 2). The RT-PCR products of circRNAs were purified for Sanger sequencing to confirm the back-spliced junction site. For circRNA quantification, quantitative real-time PCR (qPCR) was performed with specific divergent primers spanning junction site using SYBR Green Master Mix on StepOne Plus real-time PCR system (Applied Biosystems, USA). hsa_circ_0001187 and hsa_circ_0006508 were used as endogenous control in circRNA sequencing samples of LUAD and LUSC patients digested with RNase R, respectively. β-Actin was used as endogenous control for qPCR in untreated RNA samples. The primers used for qPCR were listed in Table S6 (Additional file 2).

**Statistical analysis**

All statistical differences were calculated using paired and two-sided Wilcoxon rank-sum test, and the *P* value less than 0.05 was considered as statistically significant difference. The diagnostic significance of differentially expressed circRNA was analyzed using R package plotROC (v2.2.1) [9]. All statistical analyses were conducted in the R environment (v3.5.3) (<http://www.r-project.org/>). All figures were plotted using the R package ggplot2 (v3.1.0) and GraphPad Prism (v6.01).

**References**

1. Zhang XO, Dong R, Zhang Y, Zhang JL, Luo Z, Zhang J, et al: Diverse alternative back-splicing and alternative splicing landscape of circular RNAs. Genome Res. 2016;26:1277-87.

2. Westholm JO, Miura P, Olson S, Shenker S, Joseph B, Sanfilippo P, et al: Genome-wide analysis of drosophila circular RNAs reveals their structural and sequence properties and age-dependent neural accumulation. Cell Rep. 2014;9:1966-80.

3. Gao Y, Zhang J, Zhao F: Circular RNA identification based on multiple seed matching. Brief Bioinform. 2018;19:803-10.

4. Memczak S, Jens M, Elefsinioti A, Torti F, Krueger J, Rybak A, et al: Circular RNAs are a large class of animal RNAs with regulatory potency. Nature. 2013;495:333-8.

5. Wang K, Singh D, Zeng Z, Coleman SJ, Huang Y, Savich GL, et al: MapSplice: accurate mapping of RNA-seq reads for splice junction discovery. Nucleic Acids Res. 2010;38:e178.

6. Glazar P, Papavasileiou P, Rajewsky N: circBase: a database for circular RNAs. RNA. 2014;20:1666-70.

7. Zhang XO, Wang HB, Zhang Y, Lu X, Chen LL, Yang L: Complementary sequence-mediated exon circularization. Cell. 2014;159:134-47.

8. Love MI, Huber W, Anders S: Moderated estimation of fold change and dispersion for RNA-seq data with DESeq2. Genome Biol. 2014;15:550.

9. Sachs MC: plotROC: A Tool for Plotting ROC Curves. J Stat Softw. 2017;79.
