# Supplementary material for: RNA-Seq profiling of circular RNA in human lung adenocarcinoma and squamous cell carcinoma
Source: Mol Cancer. 2019 Sep 4;18:134. doi: 10.1186/s12943-019-1061-8 (PMC6724331; doi:10.1186/s12943-019-1061-8)
Supplement: Supplementary file 2 — Table S1. Clinical characteristics of LUAD patients enrolled in this study. Table S2. Clinical characteristics of LUSC patients enrolled in this study. Table S3. The number of circRNAs produced from one gene. Table S4. Exon numbers of identified circRNAs. Table S5. The length distribution of identified circRNAs. Table S6. Information of primers used in this study. (DOCX 31 kb) [file 12943_2019_1061_MOESM2_ESM.docx]

**Table S1. Clinical characteristics of LUAD patients enrolled in this study.**

| **Number** | **Usage** | **Pathological subtype** | **Age** | **Gender** | **T** | **N** | **M** | **TNM** | **Stage** |
| --- | --- | --- | --- | --- | --- | --- | --- | --- | --- |
| 1 | circRNA-seq | LUAD | 59 | female | T1b | N0 | M0 | T1bN0M0 | IA2 |
| 2 | circRNA-seq | LUAD | 72 | female | T1b | N0 | M0 | T1bN0M0 | IA2 |
| 3 | circRNA-seq | LUAD | 64 | female | T1c | N0 | M0 | T1cN0M0 | IA3 |
| 4 | circRNA-seq | LUAD | 70 | male | T1b | N0 | M0 | T1bN0M0 | IA2 |
| 5 | circRNA-seq | LUAD | 72 | female | T1c | N0 | M0 | T1cN0M0 | IA3 |
| 6 | qPCR | LUAD | 63 | male | T1b | N0 | M0 | T1bN0M0 | IA2 |
| 7 | qPCR | LUAD | 38 | male | T2b | N2 | M0 | T2aN2M0 | IIIA |
| 8 | qPCR | LUAD | 66 | female | T1c | N0 | M0 | T1cN0M0 | IA3 |
| 9 | qPCR | LUAD | 63 | male | T1c | N1 | M0 | T1cN1M0 | IIb |
| 10 | qPCR | LUAD | 73 | female | T2a | N0 | M0 | T2aN0M0 | IB |
| 11 | qPCR | LUAD | 72 | female | T2a | N0 | M0 | T2aN0M0 | IB |
| 12 | qPCR | LUAD | 67 | male | T1c | N2 | M0 | T1cN2M0 | IIIA |
| 13 | qPCR | LUAD | 56 | female | T1b | N0 | M0 | T1bN0M0 | IA2 |
| 14 | qPCR | LUAD | 61 | male | T1b | N0 | M0 | T1bN0M0 | IA2 |
| 15 | qPCR | LUAD | 61 | female | T1b | N0 | M0 | T1bN0M0 | IA2 |
| 16 | qPCR | LUAD | 60 | female | T1b | N0 | M0 | T1bN0M0 | IA2 |
| 17 | qPCR | LUAD | 61 | female | T2a | N2 | M0 | T2aN2M0 | IIIA |
| 18 | qPCR | LUAD | 46 | female | T1c | N0 | M0 | T1cN0M0 | IA3 |
| 19 | qPCR | LUAD | 44 | female | T2a | N0 | M0 | T2aN0M0 | IB |
| 20 | qPCR | LUAD | 61 | male | T2a | N0 | M0 | T2aN0M0 | IB |
| 21 | qPCR | LUAD | 54 | female | T1c | N0 | M0 | T1cN0M0 | IA3 |
| 22 | qPCR | LUAD | 65 | female | T1b | N0 | M0 | T1bN0M0 | IA2 |
| 23 | qPCR | LUAD | 41 | male | T1c | N1 | M0 | T1cN1M0 | IIB |
| 24 | qPCR | LUAD | 62 | female | T1c | N0 | M0 | T1cN0M0 | IA3 |
| 25 | qPCR | LUAD | 59 | female | T2a | N0 | M0 | T2aN0M0 | IB |
| 26 | qPCR | LUAD | 62 | female | T1b | N0 | M0 | T1bN0M0 | IA2 |
| 27 | qPCR | LUAD | 61 | female | T1b | N0 | M0 | T1bN0M0 | IA2 |
| 28 | qPCR | LUAD | 61 | male | T2a | N0 | M0 | T2aN0M0 | IB |
| 29 | qPCR | LUAD | 50 | female | T1b | N0 | M0 | T1bN0M0 | IA2 |
| 30 | qPCR | LUAD | 49 | male | T1c | N0 | M0 | T1cN0M0 | IA3 |
| 31 | qPCR | LUAD | 58 | female | T1c | N0 | M0 | T1cN0M0 | IA3 |
| 32 | qPCR | LUAD | 89 | male | T1c | N0 | M0 | T1cN0M0 | IA3 |
| 33 | qPCR | LUAD | 72 | female | T2a | N0 | M0 | T2aN0M0 | IB |
| 34 | qPCR | LUAD | 74 | female | T1b | N0 | M0 | T1bN0M0 | IA2 |
| 35 | qPCR | LUAD | 79 | female | T1b | N0 | M0 | T1bN0M0 | IA |
| 36 | qPCR | LUAD | 71 | male | T1a | N0 | M0 | T1aN0M0 | MIA |
| 37 | qPCR | LUAD | 49 | female | T2a | N2 | M1b | T2aN0M1b | IV |
| 38 | qPCR | LUAD | 59 | male | T2a | N0 | M0 | T2aN0M0 | IB |
| 39 | qPCR | LUAD | 62 | male | T2a | N2 | M0 | T2aN2M0 | IIIA |
| 40 | qPCR | LUAD | 73 | female | T1a | N0 | M0 | T1aN0M0 | IA1 |
| 41 | qPCR | LUAD | 55 | female | T1c | N0 | M0 | T1cN0M0 | IA3 |
| 42 | qPCR | LUAD | 69 | female | T1b | N2 | M0 | T1bN2M0 | IIIA |
| 43 | qPCR | LUAD | 54 | male | T2a | N2 | M0 | T2aN2M0 | IIIA |
| 44 | qPCR | LUAD | 67 | male | T1c | N2 | M0 | T1cN2M0 | IIIA |
| 45 | qPCR | LUAD | 64 | female | T2a | N0 | M0 | T2aN0M0 | IB |
| 46 | qPCR | LUAD | 69 | male | T1c | N0 | M0 | T1cN0M0 | IA3 |
| 47 | qPCR | LUAD | 69 | male | T1b | N1 | M0 | T1bN1M0 | IIB |
|  |  |  |  |  |  |  |  |  |  |

**Table S2. Clinical characteristics of the LUSC patients enrolled in this study.**

| **Number** | **Usage** | **Pathological subtype** | **Age** | **Gender** | **T** | **N** | **M** | **TNM** | **Stage** |
| --- | --- | --- | --- | --- | --- | --- | --- | --- | --- |
| 1 | circRNA-seq | LUSC | 62 | male | T2a | N0 | M0 | T2aN0M0 | IB |
| 2 | circRNA-seq | LUSC | 55 | male | T2a | N1 | M0 | T2AN1M0 | IIB |
| 3 | circRNA-seq | LUSC | 63 | male | T2a | N0 | M0 | T2aN0M0 | IB |
| 4 | circRNA-seq | LUSC | 36 | male | T1c | N0 | M0 | T1cN0M0 | IA3 |
| 5 | circRNA-seq | LUSC | 54 | male | T2b | N0 | M0 | T2bN0MO | IIA |
| 6 | qpcr | LUSC | 68 | male | T1c | N0 | M0 | T1cN0M0 | IA3 |
| 7 | qpcr | LUSC | 64 | male | T2b | N0 | M0 | T2bN0M0 | IIA |
| 8 | qpcr | LUSC | 53 | male | T2b | N1 | M0 | T2bN1M0 | IIB |
| 9 | qpcr | LUSC | 60 | male | T1b | N0 | M0 | T1bN0M0 | IA2 |
| 10 | qpcr | LUSC | 84 | male | T1 | N0 | M0 | T1N0M0 | IA |
| 11 | qpcr | LUSC | 43 | male | T3 | N1 | M0 | T3N1MO | IIIA |
| 12 | qpcr | LUSC | 72 | male | T2a | N0 | M0 | T2aN0M0 | IB |
| 13 | qpcr | LUSC | 49 | male | T3 | N0 | M0 | T3N0MO | IIB |
| 14 | qpcr | LUSC | 61 | male | T1c | N1 | M0 | T1cN1M0 | IIB |
| 15 | qpcr | LUSC | 55 | male | T2b | N0 | MO | T2bN0M0 | IIA |
| 16 | qpcr | LUSC | 61 | male | T2 | N0 | M0 | T2N0M0 | IIA |
| 17 | qpcr | LUSC | 53 | male | T2a | N0 | M0 | T2aN0M0 | IB |
| 18 | qpcr | LUSC | 56 | male | T2a | N1 | M0 | T2aN1M0 | IIB |
| 19 | qpcr | LUSC | 44 | female | T2a | N1 | M0 | T2aN1M0 | IIB |
| 20 | qpcr | LUSC | 50 | male | T4 | N1 | MO | T4N1M0 | IIIA |
| 21 | qpcr | LUSC | 62 | male | T2a | N2 | M0 | T2aN2M0 | IIIB |
| 22 | qpcr | LUSC | 54 | male | T2a | N0 | M0 | T2aN0M0 | IB |
| 23 | qpcr | LUSC | 61 | male | T2a | N2 | M0 | T2aN2M0 | IIIB |
| 24 | qpcr | LUSC | 71 | male | T2a | N1 | M0 | T2aN1M0 | IIB |
| 25 | qpcr | LUSC | 54 | male | T3 | N2 | M0 | T3N2M0 | IIIB |
| 26 | qpcr | LUSC | 68 | male | T3 | N2 | M0 | T3N2M0 | IIIB |
| 27 | qpcr | LUSC | 64 | male | T3 | N1 | M0 | T3N1M0 | IIIA |
| 28 | qpcr | LUSC | 71 | male | T2a | N1 | M0 | T2aN1M0 | IIB |
| 29 | qpcr | LUSC | 54 | male | T4 | N0 | M0 | T4N0M0 | IIIA |
| 30 | qpcr | LUSC | 76 | female | T3 | N0 | M0 | T3N0M0 | IIB |
|  |  |  |  |  |  |  |  |  |  |

**Table S3. The number of circRNAs produced from one gene.**

| CircRNA number produced  from one gene | | Gene number | Percentage |
| --- | --- | --- | --- |
| 1 | 2,105 | | 39.20% |
| 2 | 1,029 | | 19.20% |
| 3 | 614 | | 11.40% |
| 4 | 431 | | 8.03% |
| 5 | 308 | | 5.73% |
| 6 | 207 | | 3.85% |
| 7 | 148 | | 2.76% |
| 8 | 97 | | 1.81% |
| 9 | 99 | | 1.84% |
| 10 | 66 | | 1.23% |
| >10 | 266 | | 4.95% |

**Table S4. Exon numbers of identified circRNAs.**

| Exon number of circRNA | CircRNA number | Percentage |
| --- | --- | --- |
| 1 | 483 | 2.70% |
| 2 | 4,135 | 23.10% |
| 3 | 4,669 | 26.00% |
| 4 | 3,126 | 17.40% |
| 5 | 1,958 | 10.90% |
| 6 | 1,239 | 6.91% |
| 7 | 866 | 4.83% |
| 8 | 498 | 2.78% |
| 9 | 338 | 1.89% |
| 10 | 210 | 1.18% |
| >10 | 414 | 2.31% |

**Table S5. The length distribution of identified circRNAs.**

| **Spliced length of circRNA** | | **CircRNA number** | **Percentage** |
| --- | --- | --- | --- |
| 0~200 | | 275 | 1.53% |
| 200~400 | | 6,925 | 38.60% |
| 400~600 | | 4,893 | 27.30% |
| 600~800 | | 2,454 | 13.70% |
| 800~1000 | | 1,363 | 7.59% |
| 1000~1200 | | 809 | 4.51% |
| 1200~1400 | | 462 | 2.57% |
| 1400~1600 | | 249 | 1.39% |
| 1600~1800 | | 161 | 0.90% |
| 1800~2000 | | 120 | 0.67% |
| >2000 | | 241 | 1.34% |
|  |  | |  |

**Table S6. Information of primers used in this study.**

| **Name** | **Sequence** | **Usage** | **Target name** |
| --- | --- | --- | --- |
| Forward | AAACCACCAAACACAACCTCCAAGA | qPCR | hsa_circ_0001187 |
| Reverse | GGTCTTTTTGTTTTGGTTGGCTTGT |  |  |
| Forward | TTCTGCTTTATCTGCTCCTCAAGAG | qPCR | hsa_circ_0006508 |
| Reverse | CTCCCTCCTCTTCTTTTCATTCACT |  |  |
| Forward | AGGCCAACCGCGAGAAGATG | qPCR | actin |
| Reverse | GCCAGAGGCGTACAGGGATA |  |  |
| F1 | AGCTTGTCCTTTTCCGAGC | PCR | hsa_circ_0002360 |
| R1 | GTTTCTGCCGATGTCTTCGAG |  |  |
| F2 | GTCAGATGCAGGGGAAAAGCT | qPCR |  |
| R2 | ATCCACTGTGATTTTGATGGCTCTG |  |  |
| F1 | CCTCATGGATTCTTACAGCTTGGAT | PCR | hsa_circ_0001821 |
| R1 | GATCACTGTAAATCCATCAGGCTCA |  |  |
| F2 | GCTTGAGGCCTGATCTTTTGG | qPCR |  |
| R2 | GATCACTGTAAATCCATCAGGCTCA |  |  |
| F3 | GGCTTGAGGCCTGATCTTTTG | qPCR |  |
| R3 | TGTGTCATTCCAGTGCATGGTTC |  |  |
| F1 | ACTGTCCAGTGTAAAGTGACCCTCT | PCR | hsa_circ_0077837 |
| R1 | CTCTGGATCGGAAGACTGATTCT |  |  |
| F2 | GAAAGCCCTGAGCAGAAACATAAGC | qPCR |  |
| R2 | TGGATCGGAAGACTGATTCTGCTG |  |  |
| F3 | CCAAGAAGACCAAAACTGTCCAGTG | qPCR |  |
| R3 | ACAGCTTATGTTTCTGCTCAGGGC |  |  |
|  |  |  |  |

| **Name** | **Sequence** | **Usage** | **Target name** |
| --- | --- | --- | --- |
| F1 | TTCAAATCCAGTTACACCTAAGCCA | PCR | hsa_circ_0001073 |
| R1 | ATTCTTCCAGGTAGCAAAACAATGC |  |  |
| F2 | CTGTACTTGTTCCAACTCAAGTGCTA | qPCR |  |
| R2 | TTGTCACCATAACACGGTTCAACA |  |  |
| F3 | TCAAATCCAGTTACACCTAAGCCAC | qPCR |  |
| R3 | ATCTACCAAGTATAGCACTTGAGTTGG |  |  |
| F1 | TCTAAGATTGGAGAGGTTGATGTCG | PCR | has_circ_0001495 |
| R1 | CCATCTGTCTGATTTGGTGCTTAGT |  |  |
| F2 | ATGGTGAATGGAATAATTGTGTGCC | qPCR |  |
| R2 | ATTTCCATCTGTCTGATTTGGTGCT |  |  |
| F3 | TCTAAGATTGGAGAGGTTGATGTCGA | qPCR |  |
| R3 | CACACAATTATTCCATTCACCATTATCC |  |  |

Sequences that spanning junction site within the qPCR primers are marked in grey.
