# Supplementary material for: RNA-Seq profiling of circular RNA in human lung adenocarcinoma and squamous cell carcinoma
Source: Mol Cancer. 2019 Sep 4;18:134. doi: 10.1186/s12943-019-1061-8 (PMC6724331; doi:10.1186/s12943-019-1061-8)
Supplement: Supplementary file 3 — Figure S1. Identification of circRNAs expressed in lung tumors and their adjacent normal tissues. Figure S2. Identification of differentially expressed circRNAs in LUAD and LUSC tissues. Figure S3. Validation of four selected circRNAs in LUAD and LUSC tissues. (DOCX 1305 kb) [file 12943_2019_1061_MOESM3_ESM.docx]

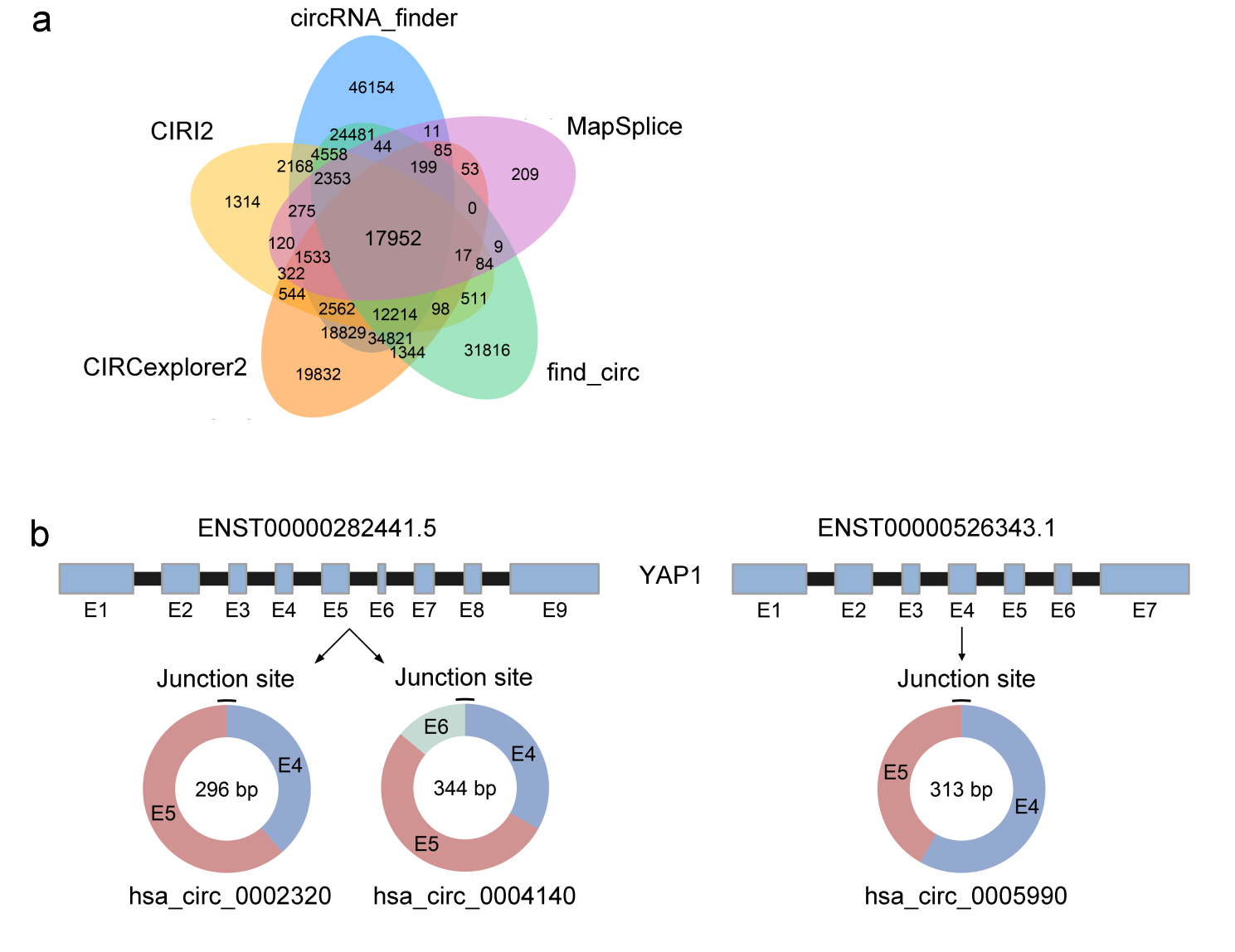


**Figure S1.** Identification of circRNAs expressed in lung tumors and their adjacent normal tissues. **a** The reliable circRNAs in lung tissues were identified by five programs. **b** Three different circRNAs, hsa_circ_0002320, hsa_circ_0004140 and hsa_circ_0005990, were produced from YAP1 gene through alternative circularization.


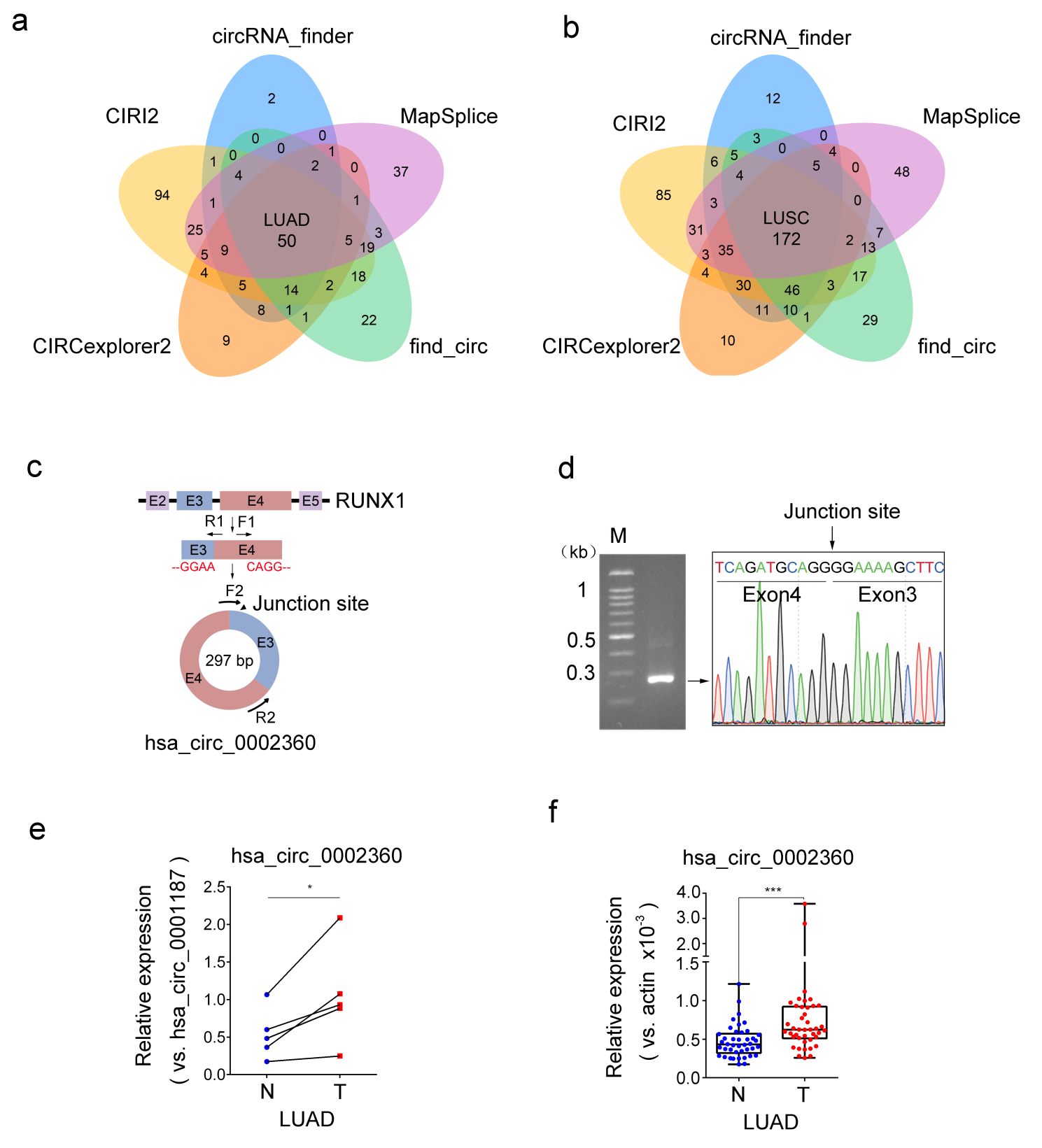


**Figure S2.** Identification of differentially expressed circRNAs in LUAD and LUSC tissues. **a-b** The differentially expressed circRNAs in LUAD (a) and LUSC (b) tissues were determined by five programs. **c** Schematic diagram of hsa_circ_0002360. **d** Agarose gel electrophoresis and Sanger sequencing of the RT-PCR products of hsa_circ_0002360. **e-f** Relative expression of hsa_circ_0002360 in LUAD tissues for circRNA sequencing (e) and in another independent cohort of NSCLC patients’ samples (f).


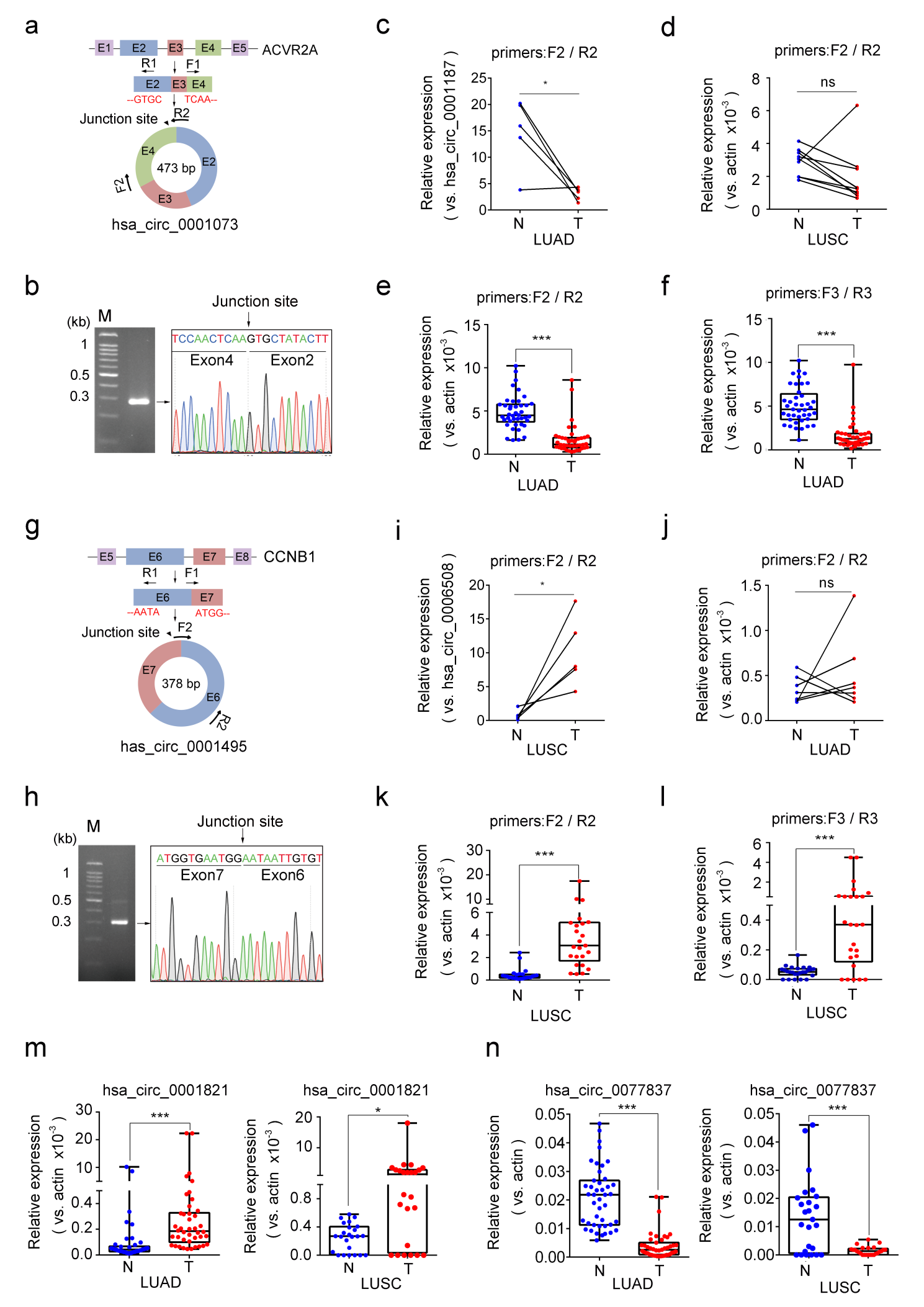


**Figure S3.** Validation of four selected circRNAs in LUAD and LUSC tissues. **a,g** Schematic diagram of hsa_circ_0001073 (a) and hsa_circ_0001495 (g). **b,h** Agarose gel electrophoresis and Sanger sequencing of the RT-PCR products of hsa_circ_0001073 (b) and hsa_circ_0001495 (h). **c-f, i-n** Relative expression of hsa_circ_0001073 (c-f), hsa_circ_0001495 (i-l), hsa_circ_0001821 (m) and hsa_circ_0077837 (n) in LUAD and LUSC tissues for circRNA sequencing and in another independent cohort of NSCLC patients’ samples.
